# Comparative analysis of Illumina, PacBio, and nanopore for 16S rRNA gene sequencing of rabbit’s gut microbiota

**DOI:** 10.3389/frmbi.2025.1587712

**Published:** 2025-05-15

**Authors:** Iliyass Biada, María Antonia Santacreu, Oscar González-Recio, Noelia Ibáñez-Escriche

**Affiliations:** ^1^ Instituto de Ciencia y Tecnología Animal, Universitat Politècnica de València, Valencia, Spain; ^2^ Instituto Nacional de Investigación y Tecnología Agraria y Alimentaria (INIA-CSIC), Madrid, Spain

**Keywords:** 16S rRNA, microbiota, Illumina, PacBio, nanopore

## Abstract

This research aims to assess whether sequencing the full length of the 16S rRNA gene using PacBio HiFi sequencing and Oxford Nanopore Technology (ONT) platforms outperform Illumina MiSeq platform in providing detailed specie level insights. Moreover, it aims to compare the similarities in microbial communities detected across the three platforms. The study employed DNA from four rabbit does’ soft feces, sequenced using Illumina MiSeq for specific 16S rRNA gene regions V3-V4, and for the complete gene sequencing using PacBio HiFi and ONT MinION. Results highlight different levels of taxonomic resolution. At the species level, PacBio and ONT exhibited the better resolutions with 63% and 76% respectively, while 48% for Illumina. However, across all three platforms, the classification output at species level was mainly labeled as “*Uncultured_bacterium*” for most of the classified sequences, which does not improve the understanding of the gut microbiota composition in rabbits. Moreover, although high correlations between relative abundances of taxa were observed, diversity analysis showed significant differences between the taxonomic compositions of the three platforms. These findings suggest that while PacBio and ONT offer improvements in species-level resolution compared to Illumina, due to references databases ambiguous annotation, all three platforms still fall short in providing a precise species level characterization of the gut microbiota composition in rabbits. Additionally, the disparities observed across the results from these platforms highlight the significant impact of sequencing platform, especially when different primers are used. This consideration is particularly important when comparing or analyzing sequences derived from different sequencing technologies.

## Introduction

The description of microbial communities within the gut microbiota is commonly achieved through the analysis of the 16S rRNA gene. Illumina is a widely used platform for amplicon sequencing of various hypervariable regions (V1-V9) of the 16S rRNA gene, providing higher number of read counts but shorter read lengths (Mosher et al., 2013). On the other hand, third-generation sequencing platforms like Pacific Biosciences (PacBio) and Oxford Nanopore Technology (ONT) offer full-length sequencing of the 16S rRNA gene, yielding longer reads. ONT improved its reads quality output up to Q20 values or higher with the new chemistries (Ferguson et al., 2022). PacBio sequencing shows nowadays high sequencing quality owing it to its Circular Consensus Sequences (CCS) protocols which provide HiFi reads with about Q27 average reads (Wenger et al., 2019).

In contrast to Illumina, these technologies possess the advantage of obtaining long reads (10^ths^ of kilobases) spanning over the full-length 16S rRNA gene, which holds promise for achieving species level taxonomic identification and facilitating a deeper understanding of the gut microbiota (Mosher et al., 2014; Santos et al., 2020). This study aims to compare the performance of Illumina, PacBio and ONT platforms to determine if the last two offer higher species level resolution. Additionally, this comparison will enable the investigation of the similarity between the microbial communities provided by these platforms, which will allow to investigate the feasibility of joint utilization of sequences from different platforms in research projects.

## Materials and methods

### Sample collection and DNA extraction

Four samples of soft feces were taken from the anus of the does by applying gentle pressure to the perianal area and immediately frozen at -72 °C until DNA extraction. Bacterial genomic DNA was isolated from the frozen fecal samples using the DNeasy PowerSoil kit (QIAGEN Inc, Hilden, Germany) following the exact protocol described in Biada et al. (2024).

### PCR amplification and DNA sequencing

The same DNA extracted from the samples was used separately in three platforms to sequence the 16S rRNA gene. First, microbial genomic DNA was amplified and purified following the 16S Metagenomic Sequencing Library Preparation protocol by Illumina (San Diego, CA, USA). The V3 and V4 regions of the 16S rRNA gene were amplified using the recommended primers (Klindworth et al., 2013). Multiplexing was performed using Nextera XT Index Kit dual indices, and the PCR products were verified with a Bioanalyzer DNA 1000 chip. Second, Pacific Biosciences (PacBio, Menlo Park, CA, USA) was used to sequence the full-length 16S rRNA gene. It was amplified using the universal primers 27F and 1492R, both tailed with PacBio barcode sequences for multiplexing. PCR amplification was performed with KAPA HiFi Hot Start DNA Polymerase over 27 cycles. Quality control was conducted using a Fragment Analyzer. The amplified DNA was pooled in equimolar concentrations, followed by library preparation with the SMRTbell Express Template Prep Kit 2.0. After assessing library quality with Qubit HS and Fragment Analyzer, sequencing was carried out on the Sequel II PacBio system using the Sequel II Sequencing Kit 2.0. Finally, for Oxford Nanopore Technologies (ONT, Oxford, UK), the 16S rRNA gene was amplified using the 16S Barcoding Kit (SQK-RAB204 and SQK-16S024) with primers 27F and 1492R, covering the full V1–V9 regions, producing ~1500 bp fragments. PCR amplification was performed using 40 cycles, with verification on an agarose gel. The PCR product was purified, quantified, and pooled equimolarly. Sequencing was conducted on a MinION device using FLO-MIN106 flow cells.

### Bioinformatic analyses

Reads from all platforms underwent quality assessment, adapter trimming, length filtering, and chimera removal. Illumina and PacBio sequences were processed using the DADA2 pipeline (Callahan et al., 2016) in R (R Core Team, 2021). PacBio’s Circular Consensus Sequencing (HiFi) generates high-fidelity reads, allowing for DADA2’s error correction and generation of ASVs. Due to the higher error rate and lack of internal redundancy in ONT, denoising with DADA2 was not feasible; instead, ONT sequences were analyzed using Spaghetti, a custom pipeline designed for processing of Nanopore 16S rRNA data, which employs an OTU-based clustering approach (Latorre-Pérez et al., 2021). High-quality reads were denoised into Amplicon Sequence Variants (ASVs) for Illumina and PacBio, while ONT reads were clustered into Operational Taxonomic Units (OTUs). Sequences from all three platforms were then imported into QIIME2 for taxonomic annotation. A Naïve Bayes classifier, trained on the SILVA database, was customized for each platform by incorporating the specific primers used for amplification and the corresponding read length distributions (Bolyen et al., 2019). After taxonomic annotation, sequences classified as Archaea, Eukaryotes, or unassigned were removed. To minimize potential artifacts, ASVs/OTUs were further filtered by excluding sequences absent in two samples or more and those with a relative abundance below 0.01%. Kendall and Pearson correlations between relative abundances and Venn diagrams (VennDiagram package) were computed in R.

### Diversity analysis

Alpha and beta diversity analyses were performed at multiple taxonomic levels, from phylum to genus, using the *phyloseq* package in R (McMurdie and Holmes, 2013). Prior to these analyses, all count tables (phylum to genus) were rarefied to an even sequencing depth. Beta diversity differences between samples and sequencing platforms were evaluated using Principal Coordinate Analysis (PCoA) based on two dissimilarity matrices, Bray-Curtis and Jaccard computed from rarefied tables. To assess the impact of sequencing platform effect and differences between individuals, a Permutational Multivariate Analysis of Variance (PERMANOVA) was performed with 10,000 permutations. To ensure robustness, beta diversity was also analyzed using a Centered Log-Ratio (CLR) transformed table based on the Aitchison dissimilarity matrix. Principal Component Analysis (PCA) was then computed, and a second PERMANOVA test (10,000 permutations) was used to assess differences between individuals and methods based on Aitchison distances. Alpha diversity analysis was assessed using Kruskal-Wallis test based on three alpha diversity indices measured from rarified tables: Pielou evenness, observed richness diversity and Shannon diversity.

## Results

After quality filtering, the average number of reads per sample was 30,184 ± 1,146 (0.12 gigabases) for Illumina, 41,326 ± 6,174 for PacBio (0.55 gb), and 630,029 ± 92,449 (0.89 gb) for ONT. Illumina paired-end reads had an average length of 442 ± 5 base pairs (bp), while PacBio and ONT produced single-end reads with average lengths of 1,453 ± 25 bp and 1,412 ± 69 bp, respectively. Following quality control and filtering of potential artifacts, the ASVs identified was 725 for Illumina and 998 for PacBio. For ONT, a total of 923 Operational Taxonomic Units (OTUs) were identified.

**Figure 1:**
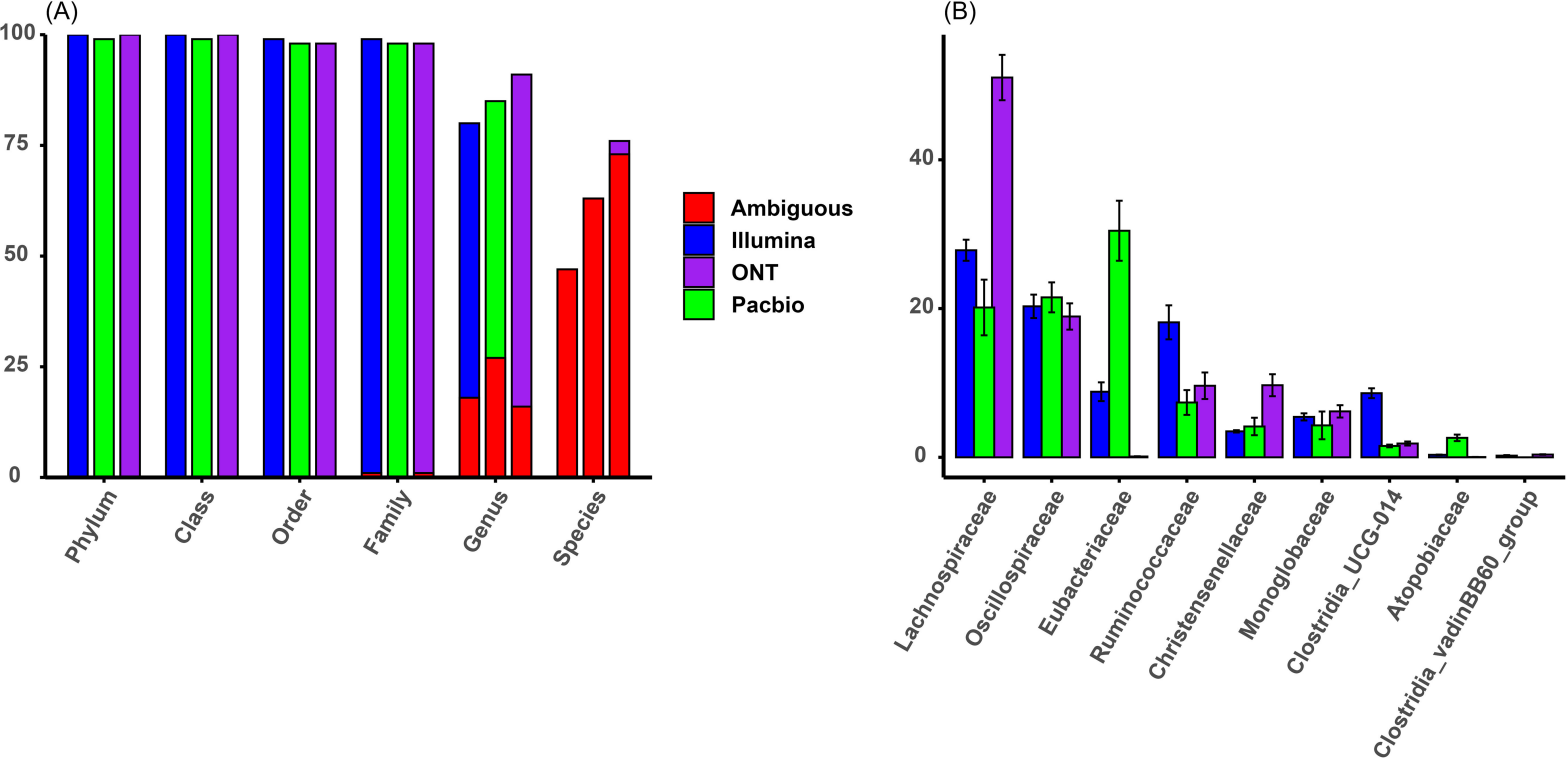
**(A)** Taxonomic classification resolution across sequencing platforms and taxonomic levels in percentages (section of bars colored in red indicate sequences classified with ambiguous names). **(B)** Taxonomic composition at family level across sequencing platforms in relative abundance.

The taxonomic resolution results for Illumina, PacBio, and ONT sequencing platforms are shown in **Figure 1A**. All three platforms achieved similar resolution up to the family level, classifying at least 99% of sequences (1% unidentified). However, differences emerged at the genus and species levels. ONT performed best, classifying 91% of sequences to genus level and 76% to species level. PacBio followed, with 85% classified to genus level and 63% to species level. Illumina had the lowest resolution, classifying 80% of sequences to genus level and 47% to species level. At the species level, ONT classified 29% more sequences than Illumina, while PacBio classified 16% more. However, as highlighted in red in **Figure 1A**, most sequences classified to species level were uncultured and were assigned ambiguous names, such as *uncultured_bacterium*, indicating limited reliable species-level identification.

The comparison of relative abundances across the three platforms showed differences in how consistently microbial families were detected and quantified, as shown in **Figure 1B**. The most abundant families, including *Lachnospiraceae*, *Oscillospiraceae*, *Eubacteriaceae*, and *Ruminococcaceae*, were found in all platforms, but their relative abundances varied. For example, *Lachnospiraceae* was most dominant in ONT (51.06% ± 6.10%), with nearly double the abundance compared to Illumina (27.84% ± 2.84%) and PacBio (20.14% ± 7.48%). *Oscillospiraceae* had stable abundances across platforms (Illumina: 20.30% ± 3.15%; ONT: 18.93% ± 3.53%; PacBio: 21.51% ± 4.03%). In contrast, *Eubacteriaceae* was much more abundant in PacBio (30.46% ± 8.07%) than in Illumina (8.82% ± 2.52%) and was almost absent in ONT (0.11% ± 0.07%). *Ruminococcaceae* also showed variability, with the highest abundance in Illumina (18.15% ± 4.57%), followed by ONT (9.62% ± 3.57%) and PacBio (7.37% ± 3.34%). Similar inconsistencies were seen in lower abundance families like *Christensenellaceae* and *Clostridia_UCG-014*, while others, such as *Monoglobaceae*, were more consistent across platforms. These results highlight that different sequencing platform can influence the observed microbial community composition.

The strength of relationships between relative abundances of the identified taxa by Illumina, PacBio, and ONT was assessed. Both Kendall and Pearson correlation coefficients, were employed and results are summarized in **Table 1**. Pearson correlations were consistently high at broader taxonomic levels, with perfect agreement (Pearson = 1.00) observed at the phylum and class levels across all platform comparisons. However, these correlations declined at finer resolutions, with the genus level showing the weakest values. For instance, Illumina-PacBio at 0.77, Illumina-ONT at 0.52, and PacBio-ONT at 0.34 (**Table 1**). Kendall correlations showed a similar trend but were generally lower than Pearson correlations. At the phylum level, a strong agreement was observed for Illumina-PacBio and PacBio-ONT (Kendall = 0.87 and 0.82, respectively), while correlations between ONT and Illumina were more moderate (0.62). At finer taxonomic resolutions, such as the genus level, Illumina-PacBio maintained relatively higher correlations (Kendall = 0.59), while Illumina-ONT and PacBio-ONT showed weaker agreement (0.40 and 0.45 respectively).

**Table 1:** Correlation comparison between sequencing platforms Illumina, PacBio and Oxford Nanopore Technology (ONT) at different taxonomic levels.

Taxonomic level	Illumina-PacBio		Illumina-ONT		PacBio-ONT	
	Kendall	Pearson	Kendall	Pearson	Kendall	Pearson
Phylum	0.84	1.00	0.62	1.00	0.82	1.00
Class	0.74	1.00	0.49	1.00	0.73	1.00
Order	0.74	0.81	0.52	0.86	0.54	0.62
Family	0.65	0.77	0.53	0.87	0.53	0.59
Genus	0.59	0.77	0.40	0.52	0.45	0.34

**Figure 2:**
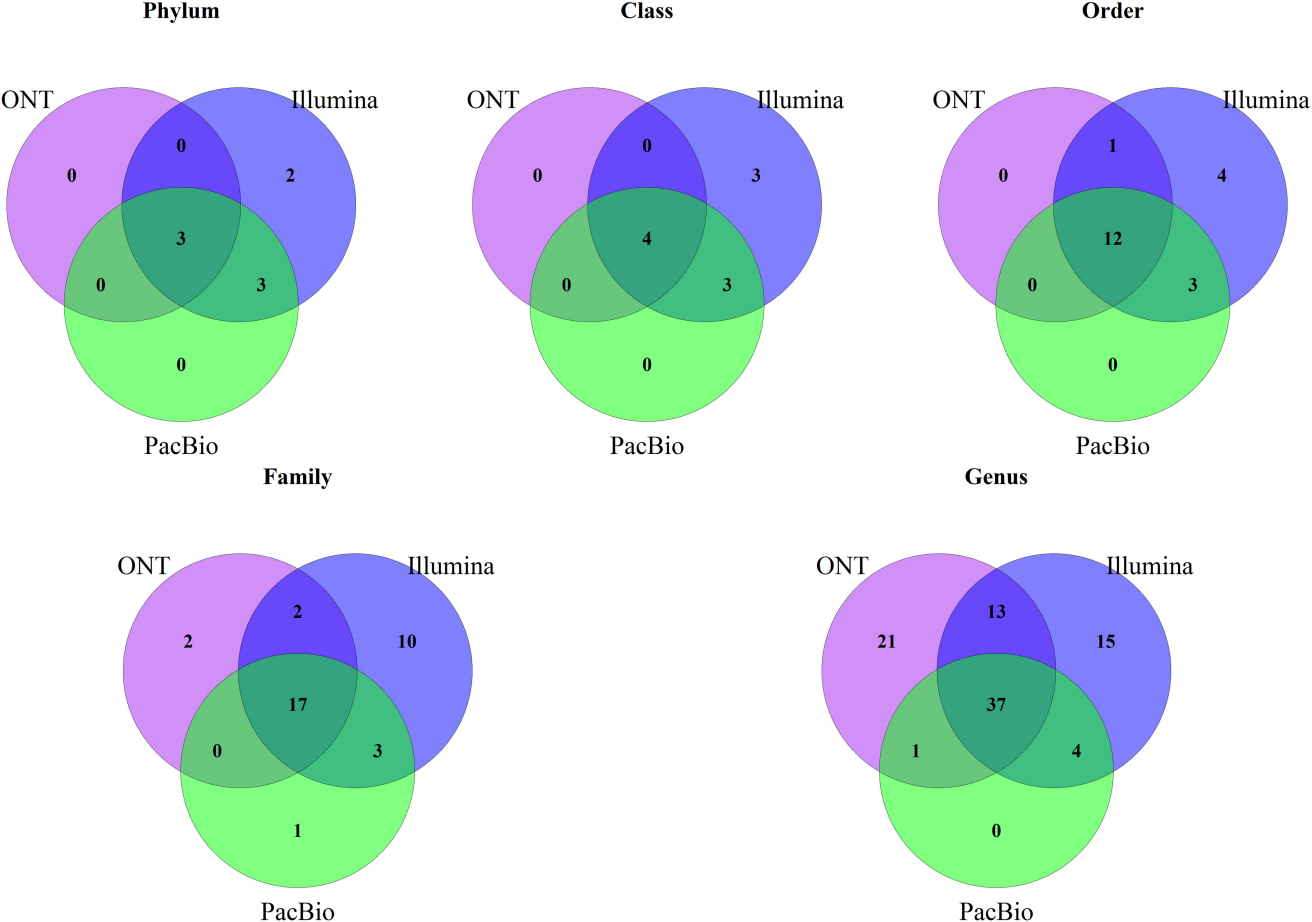
Venn diagram of shared taxa between the three platforms, Illumina, PacBio and Oxford Nanopore Technology (ONT) at different taxonomic levels.

After comparing relative abundances across sequencing platforms, we analyzed the identified taxa by assessing their presence or absence and conducting alpha and beta diversity analyses. First, we used Venn diagrams (**Figure 2**) to compare taxa presence across platforms. At the phylum, class, and order levels, Illumina was the only platform that detected unique taxa to it. For example, at the phylum level, Illumina uniquely identified *Desulfobacterota* and *Proteobacteria*. Differences were more pronounced at the family and genus levels. Across all platforms, 37 genera were shared, while Illumina detected 15 unique genera and ONT detected 21. Notably, PacBio did not detect any unique taxa, except at the family level.

**Figure 3:**
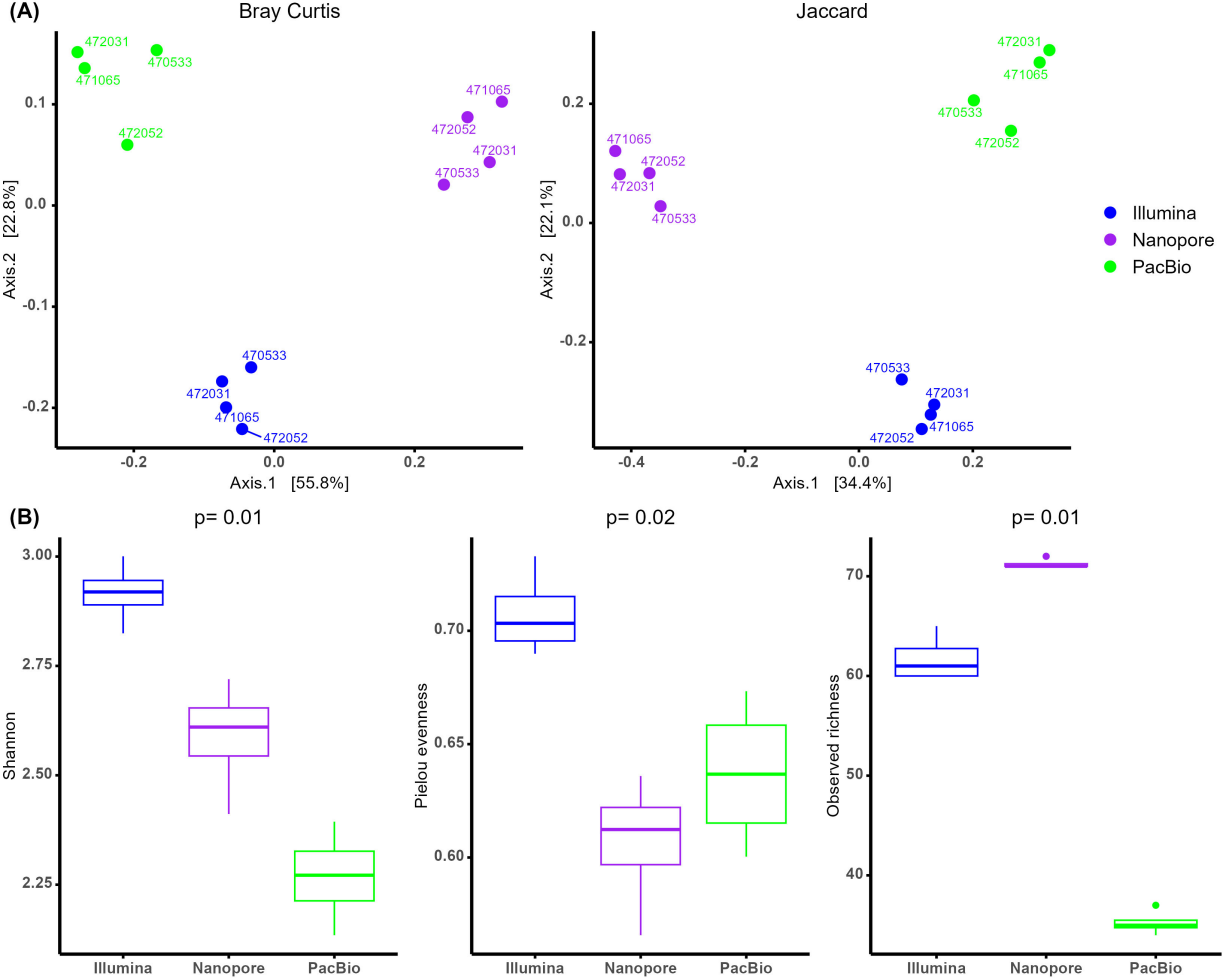
**(A)** Principal Coordinate Analysis (PCoA) of beta diversity at genus level based on Bray-Curtis (left) and Jaccard (right) distance matrices, with samples colored by sequencing method. **(B)** Alpha diversity boxplots of the comparison between sequencing platforms at genus level.

The diversity analyses further confirmed the differences observed among the sequencing platforms. Both beta and alpha diversity analyses revealed variations across all taxonomic levels (**Supplementary File 1**). Here, we present the results at the genus level. Beta diversity analysis using PERMANOVA showed significant differences between the three sequencing platforms, with variation detected for both Bray-Curtis (p < 0.001, R² = 0.76) and Jaccard (p < 0.001, R² = 0.67) distances. PCoA plots further supported these distinctions, with the first and second PCoA axes explaining 79% and 56% of the variance for Bray-Curtis and Jaccard distances, respectively (**Figure 3A**). In contrast, differences between females were not statistically significant (Bray-Curtis: p = 0.19, R² = 0.04 and Jaccard: p = 0.24, R² = 0.05), suggesting that while the sequencing platform had a strong influence on microbial community composition, variation between individuals had a minor impact on the observed diversity patterns. Beta diversity was also analyzed using CLR transformed table instead of rarefication, and PERMANOVA and PCA analyses results using Aitchison distance were the same as Bray-Curtis and Jaccard (**Supplementary File 1**).

Alpha diversity analysis also revealed significant differences across the three indices (**Figure 3B**). Shannon diversity, which implements both richness and evenness revealed higher values for Illumina, followed by ONT and finally PacBio. Concerning evenness results, Illumina maintained the highest values, however in observed richness ONT showed the highest values.

## Discussion

Several studies have reported that third-generation full-length 16S rRNA sequencing technologies, such as PacBio and ONT, generally achieve higher classification resolution at the species level in comparison to Illumina (Buetas et al., 2024; Mosher et al., 2014; Nygaard et al., 2020; Santos et al., 2020; Shin et al., 2016; Szoboszlay et al., 2023). Our findings align with this trend, as both PacBio and ONT outperformed Illumina in species-level taxonomic resolution. The increase observed in our study was important, with PacBio and ONT achieving 16% and 29% higher resolution respectively, compared to Illumina. However, upon thorough examination of the species identified across all platforms, it becomes evident that almost all of sequences were annotated ambiguously in the species level, across all platforms (for example as “*Uncultured_bacterium*”, “*gut_metagenome*” and others), and only few sequences were annotated with the name of bacterial species (**Supplementary File 2**). Unfortunately, this nomenclature does not enhance our understanding of the specie level gut community of rabbits as expected. This observation strongly emphasizes the ongoing need for refinement within the reference databases, which are crucial in facilitating accurate taxonomic assignments, especially in rabbits.

The results of comparisons of relative abundances revealed differences in the detection and quantification of microbial taxa across sequencing platforms. While some families, such as *Oscillospiraceae*, showed stable abundances across platforms, others, like *Lachnospiraceae, Eubacteriaceae*, and *Ruminococcaceae*, exhibited substantial variability. For instance, *Lachnospiraceae* was dominant in ONT but showed much lower abundances in Illumina and PacBio. Similarly, *Eubacteriaceae* was highly abundant in PacBio but nearly absent in ONT. Other studies have reported similar results with differences in relative abundances from different sequences platforms at different taxonomic levels (Buetas et al., 2024; Yeo et al., 2024). The correlation analysis showed strong agreement at higher taxonomic levels (phylum/class) but decreased at finer resolutions. Pearson correlations, which measure linear relationships remained high broadly but declined at the genus level, with PacBio-ONT showing the weakest agreement. In terms of ranking agreement, measured by Kendall correlations, a similar trend was observed but values were generally lower. These findings align with previous studies, which report strong platform agreement at higher taxonomic levels but variability at lower taxonomic levels (Karst et al., 2021; Nygaard et al., 2020; Yu et al., 2015).

We should note that PacBio and Illumina had slightly higher correlations when compared to ONT, this can be because of a bias of the methodology used, since PacBio and Illumina were both analyzed using DADA2 pipeline which gives ASVs through denoising, while this step was not performed in the case of ONT, and sequences were clustered into OTUs. ASVs provide single-nucleotide resolution and better reproducibility, while OTUs cluster sequences at a similarity threshold (commonly 97%), potentially merging distinct taxa (Callahan et al., 2016). This difference in resolution could contribute to the slightly lower correlation and higher richness observed in ONT. Therefore, part of the observed discrepancies might not only stem from sequencing technology, but also from the bioinformatics employed.

Our study also analyzed presence or absence of taxa in sequencing platforms, identifying several taxa that were only identified by Illumina, and to a lesser extent by ONT, especially at finer taxonomic levels (family and genus). Notably, these taxa exclusive to individual platforms were generally present in low relative abundances. For example, at the genus level, Illumina detected 69 genera, PacBio identified 42, and ONT found 72 (**Figure 1A**). Of these, 37 genera were shared across all platforms, collectively accounting for the majority of the relative abundance: 94% in Illumina, 96% in PacBio, and 93% in ONT. This explains the relatively high correlations observed between platforms, because taxa with very low relative abundances (often exclusive to a single platform) had a minimal effect on reducing overall correlations.

Beta diversity comparisons, which account for low-abundance taxa, provided a clearer distinction between sequencing platforms. Both PCoA and PERMANOVA analyses showed significant differences in community structure depending on the platform. Similar beta diversity differences were observed between Illumina and ONT (Yeo et al., 2024). When comparing Illumina and PacBio, one study reported differences in beta diversity using phylogenetic distances (Unifrac) (Katiraei et al., 2022). However, Buetas et al. (2024) found no significant beta diversity differences between Illumina and PacBio when using Bray-Curtis distances. Alpha diversity analyses also varied across platforms. Illumina exhibited higher Shannon diversity values, which account for both richness and evenness, compared to ONT and PacBio. Similar trends have been observed in previous studies comparing Illumina and PacBio (Buetas et al., 2024; Wagner et al., 2016). Although ONT had lower Shannon diversity values than Illumina, it demonstrated higher observed richness, which reflects absolute species counts without considering their relative abundances. The literature presents mixed findings for ONT: some studies, such as Stevens et al. (2023), align with our results, reporting greater richness compared to Illumina, while others, like Heikema et al. (2020), found no significant differences between platforms.

The differences between platforms found in this study suggest that the choice of sequencing platform, which not only determines read length but also introduces biases through primer selection and amplification efficiency, can significantly influence the observed microbial community composition. This has important implications for microbiome research, particularly when comparing results across studies using different sequencing technologies. Therefore, we strongly advise against comparing microbiome results from studies using different sequencing technologies, as this practice may lead to inaccurate interpretations and erroneous conclusions. Additionally, for researchers prioritizing detailed species-level resolution, PacBio or ONT are recommended due to their superior taxonomic resolution compared to Illumina. Nevertheless, until species-level reference databases are further refined, Illumina remains a cost-effective choice for studies aiming to assess overall microbial community structure and diversity.

It is also important to acknowledge the limitations of this study, particularly the small sample size of only four samples and the absence of a mock community with known microbial compositions for validation. These factors necessitate caution when generalizing our findings to a broader population or other species. Despite these limitations, this study provides valuable insights by focusing on rabbits, an understudied species in microbiome research. It highlights the challenges associated with current sequencing platforms and underscores the need for more refined, species-specific databases for rabbit microbiomes, ultimately contributing to a more accurate understanding of rabbit gut microbiota.

## Conclusion

This study confirmed that full length 16S rRNA sequencing using third-generation platforms, particularly PacBio and ONT, achieve higher taxonomic resolution at the species level compared to Illumina. However, despite this increased resolution, species-level classification remains unreliable due to limitations in existing reference databases. This finding highlights the need for improved reference databases, particularly for rabbit microbiomes, to facilitate more precise understanding of rabbit gut microbiota compositions at the species level. Additionally, despite broad similar compositions of relative abundance at higher taxonomic levels, significant disparities in the identified taxa and diversity emerged when comparing the three platforms. Given these inconsistencies, taxonomic results from different sequencing platforms should not be directly compared or aligned without careful consideration of methodological biases.

## Data Availability

The original contributions presented in the study are publicly available. This data can be found here: NCBI Sequence Read Archive (SRA), accession PRJNA1245308.
