# Supplementary material for: Comparative analysis of Illumina, PacBio, and nanopore for 16S rRNA gene sequencing of rabbit’s gut microbiota
Source: Front Microbiomes. 2025 May 15;4:1587712. doi: 10.3389/frmbi.2025.1587712 (PMC12993484; doi:10.3389/frmbi.2025.1587712)
Supplement: Supplementary file 2 [file SupplementaryFile1.docx]

Supplementary Material


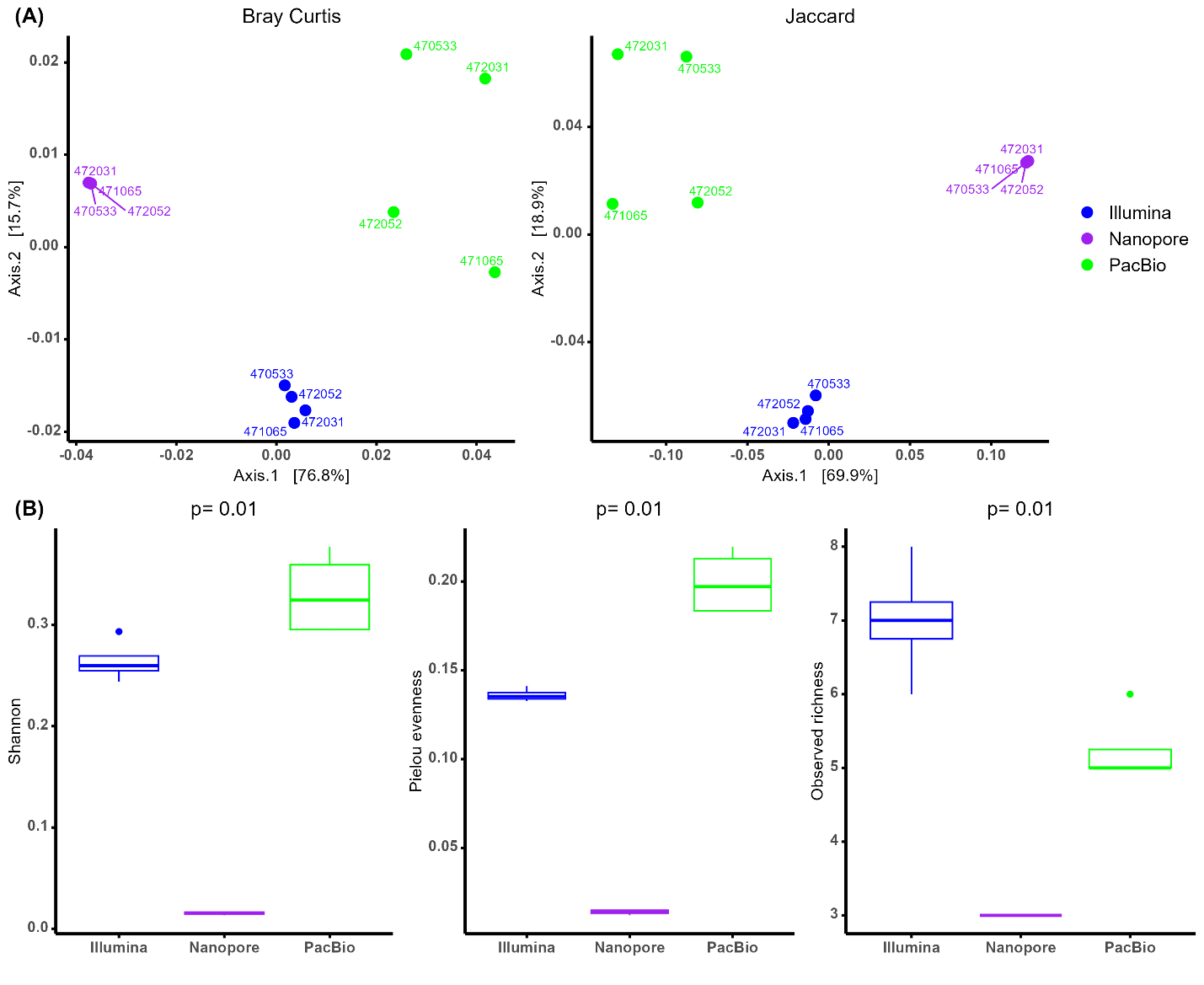


**Figure S1. A.** Principal Coordinate Analysis (PCoA) of beta diversity at phylum level based on Bray-Curtis (left) and Jaccard (right) distance matrices, with samples colored by sequencing method. **B.** Alpha diversity boxplots of the comparison between sequencing platforms at phylum level.


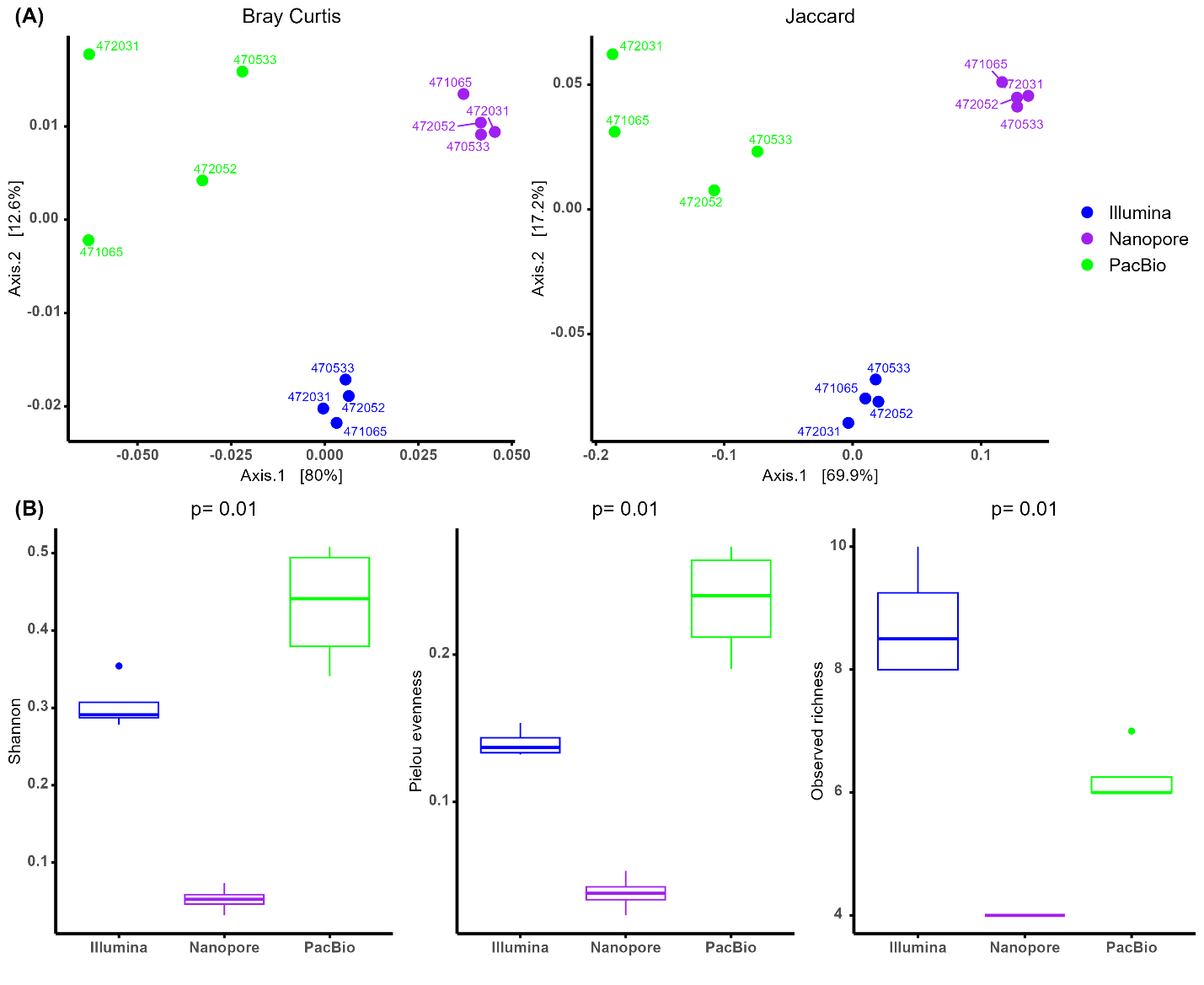


**Figure S2. A.** Principal Coordinate Analysis (PCoA) of beta diversity at class level based on Bray-Curtis (left) and Jaccard (right) distance matrices, with samples colored by sequencing method. **B.** Alpha diversity boxplots of the comparison between sequencing platforms at class level.


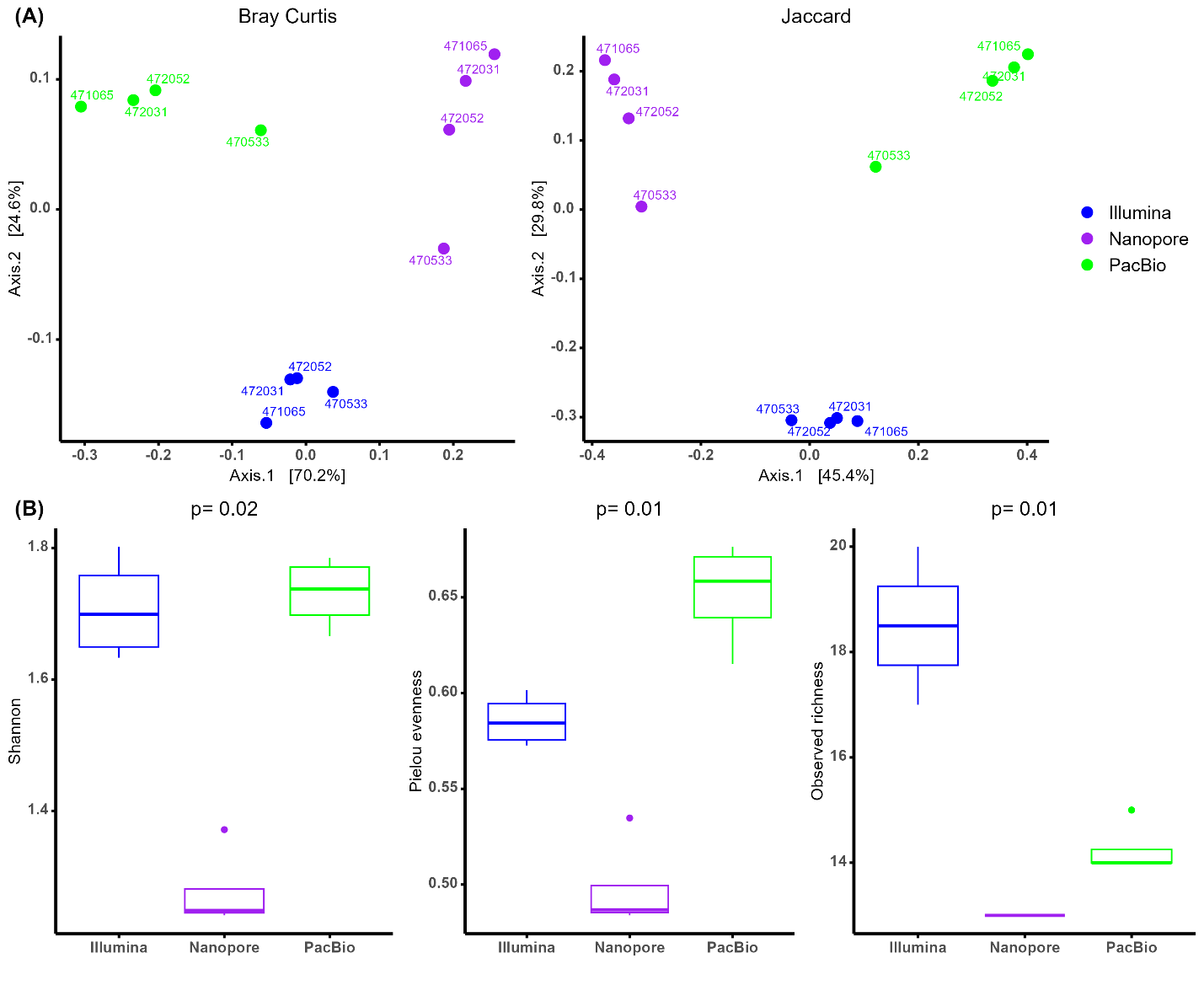


**Figure S3. A.** Principal Coordinate Analysis (PCoA) of beta diversity at order level based on Bray-Curtis (left) and Jaccard (right) distance matrices, with samples colored by sequencing method. **B.** Alpha diversity boxplots of the comparison between sequencing platforms at order level.


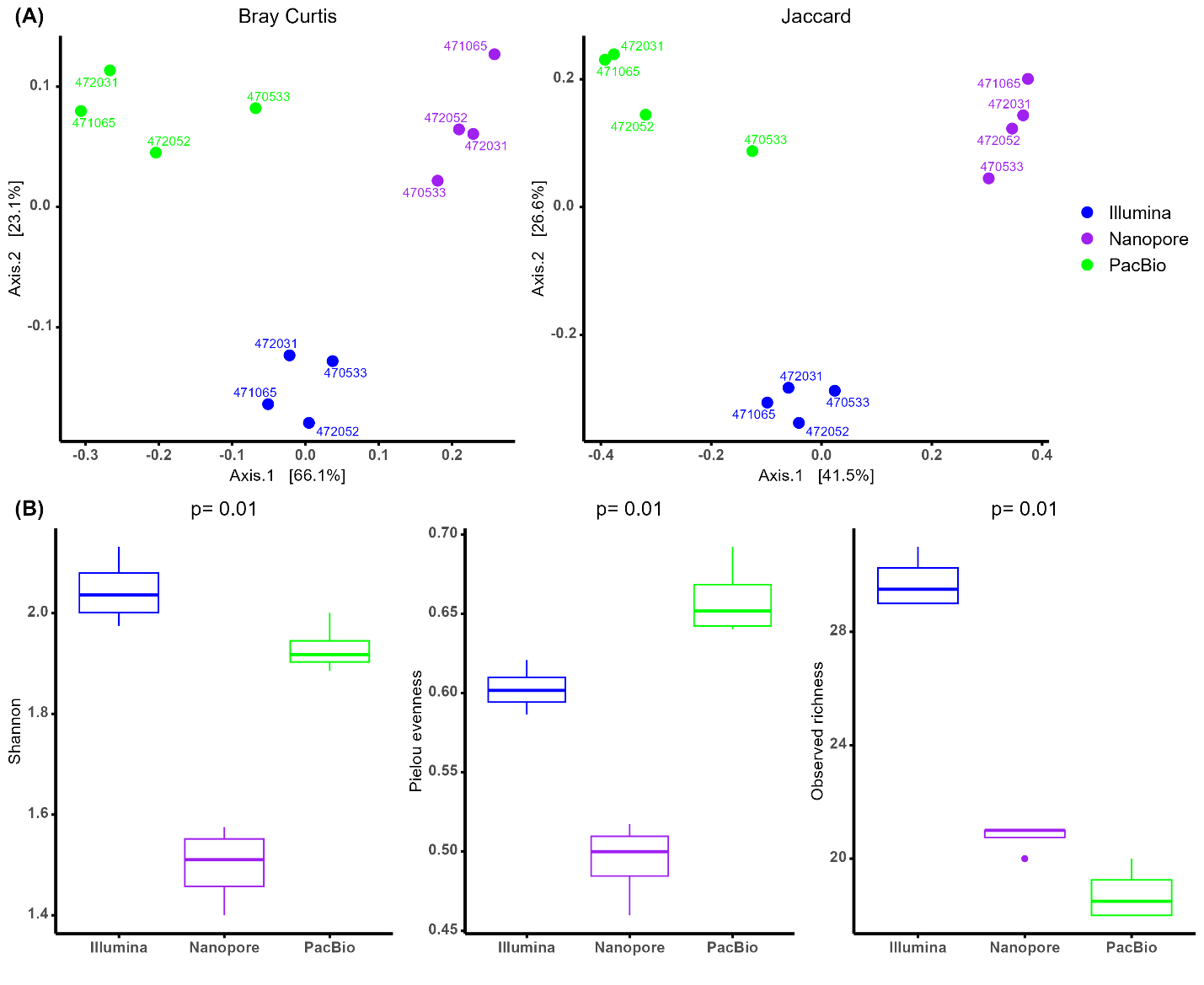


**Figure S4. A.** Principal Coordinate Analysis (PCoA) of beta diversity at family level based on Bray-Curtis (left) and Jaccard (right) distance matrices, with samples colored by sequencing method. **B.** Alpha diversity boxplots of the comparison between sequencing platforms at family level.


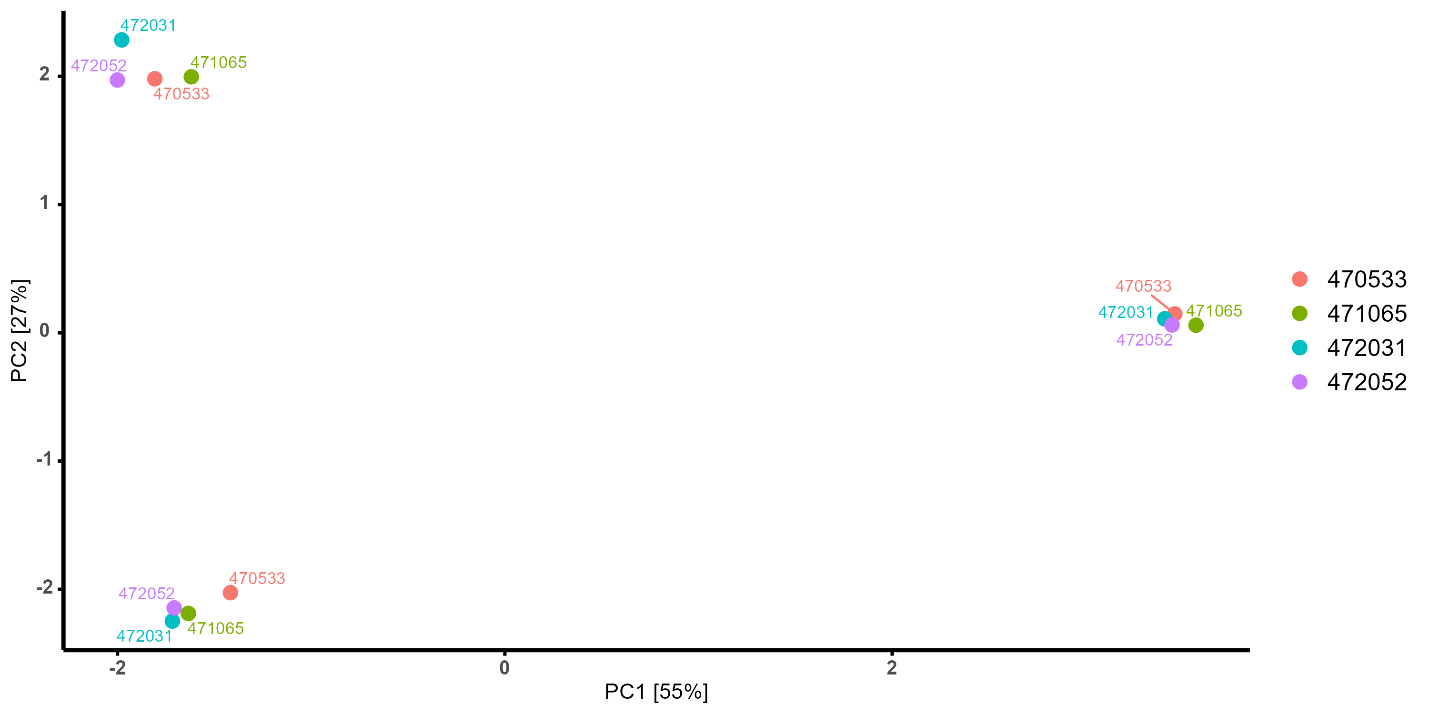


**Figure S5.** Principal Coordinate Analysis (PCoA) of beta diversity at genus level based on Aitchison distance matrices, with samples colored by female.
